# MicroRNA-30b-Mediated Regulation of Catalase Expression in Human ARPE-19 Cells

**DOI:** 10.1371/journal.pone.0042542

**Published:** 2012-08-06

**Authors:** Rashidul Haque, Eugene Chun, Jennifer C. Howell, Trisha Sengupta, Dan Chen, Hana Kim

**Affiliations:** Department of Ophthalmology, Emory University School of Medicine, Atlanta, Georgia, United States of America; University of Florida, United States of America

## Abstract

**Background:**

Oxidative injury to retinal pigment epithelium (RPE) and retinal photoreceptors has been linked to a number of retinal diseases, including age-related macular degeneration (AMD). Reactive oxygen species (ROS)-mediated gene expression has been extensively studied at transcriptional levels. Also, the post-transcriptional control of gene expression at the level of translational regulation has been recently reported. However, the microRNA (miRNA/miR)-mediated post-transcriptional regulation in human RPE cells has not been thoroughly looked at. Increasing evidence points to a potential role of miRNAs in diverse physiological processes.

**Methodology/Principal Findings:**

We demonstrated for the first time in a human retinal pigment epithelial cell line (ARPE-19) that the post-transcriptional control of gene expression via miRNA modulation regulates human catalase, an important and potent component of cell's antioxidant defensive network, which detoxifies hydrogen peroxide (H_2_O_2_) radicals. Exposure to several stress-inducing agents including H_2_O_2_ has been reported to alter miRNA expression profile. Here, we demonstrated that a sublethal dose of H_2_O_2_ (200 µM) up-regulated the expression of miR-30b, a member of the miR-30 family, which inhibited the expression of endogenous catalase both at the transcript and protein levels. However, antisense (antagomirs) of miR-30b was not only found to suppress the miR-30b mimics-mediated inhibitions, but also to dramatically increase the expression of catalase even under an oxidant environment.

**Conclusions/Significance:**

We propose that a microRNA antisense approach could enhance cytoprotective mechanisms against oxidative stress by increasing the antioxidant defense system.

## Introduction

The RPE, located between the light sensitive outer segments of the photoreceptors and the blood supply of the choroid, is required for light absorption, epithelial transport, retinoid recycling, and removal of molecular components that shed from the photoreceptor outer segment in order to have continued vision [1]. The RPE is at high risk for oxidative stress because it is exposed to high levels of phototoxic blue light and high oxygen tension [2]–[4]. Among the ROS to which the cells are exposed is H_2_O_2_. As in most cells, H_2_O_2_ is generated during normal oxygen metabolism in mitochondria. In the RPE, H_2_O_2_ is also produced during daily phagocytosis of shed photoreceptor outer segments [5], [6] and is generated as a consequence of light irradiation of the pigment melanin [7]. It has been reported that the phagocytosis-generated H_2_O_2_ causes mitochondrial dysfunction and damage mitochondrial DNA in human RPE cells [8]. Therefore, accumulated oxidative damage in the largely non-mitotic RPE monolayer is likely to cause tissue dysfunction that may contribute to the pathogenesis of retinal diseases, including AMD [9]–[11].

ROS such as H_2_O_2_ that indiscriminately attack DNA, proteins, lipids, and other cellular components play an important role in controlling cellular functions such as cell differentiation, proliferation, migration, apoptosis, and death [12], [13]. To combat the deleterious effects of ROS, cells have evolved an intrinsic antioxidant defense network that consists of a variety of scavengers, such as superoxide dismutases (SODs), glutathione peroxidases (GPx-s), catalase, and glutathione S-transferases (GSTs). Protection against superoxide radicals is provided by SODs, which catalyze the dismutation of superoxide to H_2_O_2_ [14]. Catalase catalyzes the conversion of H_2_O_2_ to water (preventing the generation of hydroxyl radicals) and oxygen, and GPx-s catalyze the reduction of H_2_O_2_ and reduce lipid hydroperoxides to their corresponding alcohols and free H_2_O_2_ to water [15]. Catalase has the highest turnover number of all antioxidant enzymes: one molecule of catalase can convert more than 6 million molecules of H_2_O_2_ to H_2_O and O_2_ each minute [16]. In addition to enzymatic scavengers, nonenzymatic molecules, such as ascorbate, tocopherol, glutathione, and carotinoids are also important [14].

Microarray analysis done by Weigel et al. [13] and Vandenbroucke et al. [17] revealed that a huge number of genes regulated in cells treated with H_2_O_2_ are responsible for H_2_O_2_-mediated cellular effects. Transcriptional regulation in H_2_O_2_-mediated oxidative stress has been shown by many investigators [12], [13], [17]. However, the post-transcriptional mechanism of gene expression in response to H_2_O_2_-mediated oxidative stress in RPE cells has not been thoroughly looked at. One such translational mechanism that regulates a large number of developmental and physiological processes is accomplished by miRNA. MicroRNAs are evolutionarily conserved, small (approx. 19 to 25 nt), single-stranded, non-protein coding RNA molecules that recognize sequences in the 3′-untranslated regions (3′-UTR) of target messenger RNAs, and induce either mRNA degradation [18] or inhibit translation of proteins [19], [20]. It is currently estimated that the human genome may have 800–1,000 miRNAs [21].

Recently, miRNAs have been shown to be directly linked to retinal diseases like retinitis pigmentosa [22], retinoblastoma [23], and ocular neovascularization [24], [25]. Although miRNAs represent a new layer of gene expression regulators at the post-transcriptional level, the effects of ROS on miRNA expression and the potential roles of miRNAs in ROS-mediated gene regulation and biological functions of retinal and RPE cells are unclear. ARPE-19, a cell line derived from human RPE, has been widely used to investigate the response of RPE to oxidative stress [26]–[28].

The computational analysis predicted a number of miRNAs including miR-30b that could putatively target catalase mRNA. Our *in silico* analysis also found that the miR-30b could potentially target genes such as integrin beta 3 (ITGB3), C-reactive protein (CRP), paraoxonase 2 (PON2), retinoblastoma 1(RB1), retinitis pigmenstosa (RP) GTPase regulator (RPGR), and endothelin receptors (EDNR) that are likely candidates of oxidative stress-mediated ocular diseases such as AMD, diabetic retinopathy (DR), glaucoma, RP, and retinoblastoma. Therefore, we decided to examine if miR-30b could potentially target and regulate the expression of catalase, a major enzyme in the intracellular antioxidant defense mechanism and a key scavenger of H_2_O_2_. Here, we found that miR-30b negatively regulated catalase expression in ARPE-19 cells and a microRNA antisense approach increased catalase expression by suppressing the miR-30b-mediated inhibition of catalase expression under oxidative stress.

## Results

### Mir-30b interacts with the 3′-UTR of the human catalase mRNA

**Figure 1:**
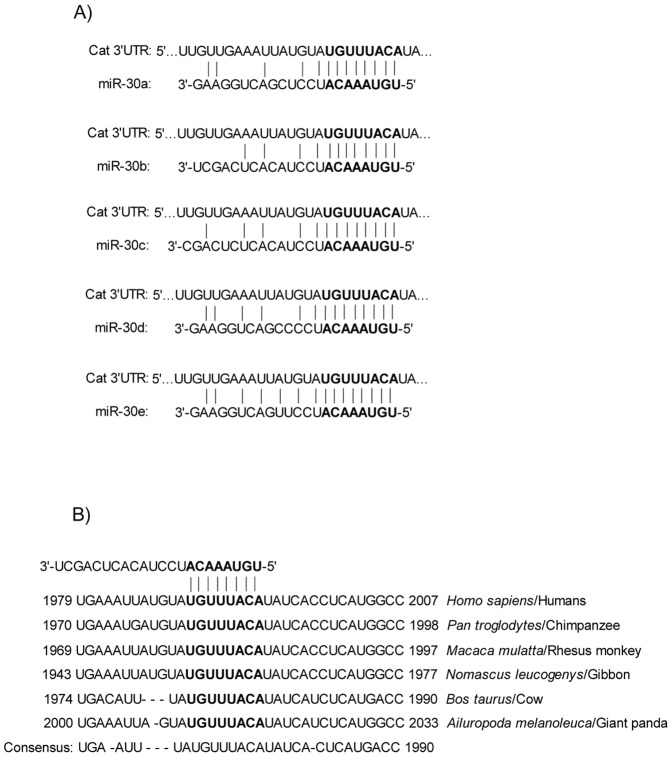
The human catalase 3′-UTR contains one putative miRNA binding site for the members of miR-30 family. Panel A: Complimentarity between the members of miR-30 family and the putative human catalase 3′-UTR site targeted (318–324 bp downstream from the human catalase stop codon). Panel B: The potential binding sequences for miR-30b within the catalase 3′-UTR of human (*H. sapiens*), chimpanzee (*P. troglodytes*), rhesus monkey (*M. mulatta*), gibbon (*N. leucogenys*), cow (*B. taurus*), and giant panda (*A. melanoleuca*). The 8 bp seed sequences of miR-30 and the putative target site in catalase 3′-UTR in both the panels are highlighted in bold.

Using TargetScan and miRanda algorithms, the human catalase was predicted to be the putative target of miR-30. *In silico* analysis of these databases demonstrated that the human catalase 3′-UTR harbors a single binding site for the members of miR-30 family. For this study we have focused on miR-30b that may target the human catalase 3′-UTR since this site is conserved across species. The bioinformatic analysis for the target site of miR-30 in catalase 3′-UTR is shown in **Figure 1**.

### Effects of H_2_O_2_ on cell viability and protein carbonylation

**Figure 2:**
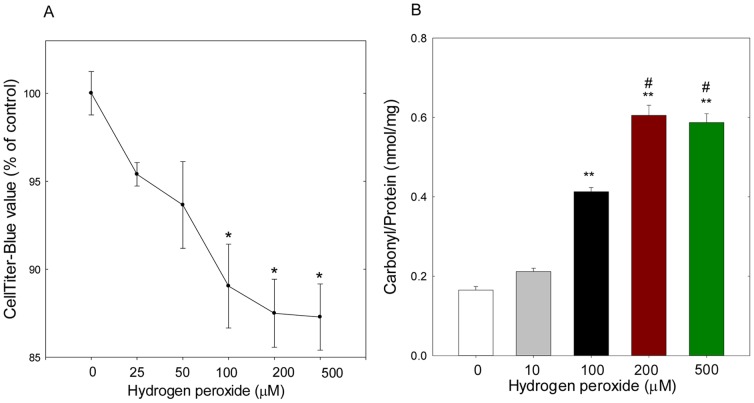
Cell viability (A) and protein carbonylation (B) in H_2_O_2_-treated ARPE-19. ARPE-19 cells were treated with various doses of H_2_O_2_ for 18 h and assessed for cell viability (A) and protein carbonylation (B). Data were analyzed using analysis of variance (ANOVA) with Student–Newman–Keuls multiple comparison tests. Data are expressed as a percentage of the untreated control. n = 5, mean ± SEM, *p = 0.002 vs. control, **p<0.001 vs. control, #*p*<0.001 vs. 100 µM.

Addition of H_2_O_2_ to the medium of cultured cells has been used as a technique to assess aspects of the oxidative defense system in RPE cells. Before examining the effect of H_2_O_2_-mediated oxidative stress on catalase and miRNA expression in ARPE-19 cells, the viability of ARPE-19 cells was determined using the CellTiter-Blue assay by exposing the cells to 25, 50, 100, 200, and 500 µM H_2_O_2_ for 18 h. Cells treated with vehicle (0 µM H_2_O_2_) were maintained as control. The CellTiter-Blue viability assay showed that H_2_O_2_ induced a gradual reduction in CellTiter-Blue values in a dose dependent manner after 18 h treatment under our experimental conditions as mentioned in ‘Materials and Methods’. H_2_O_2_ at low concentrations (<100 µmol/L) gradually reduced cell viability, but at higher concentrations (>100 µmol/L), a significant reduction (*p*<0.05) of cell viability was detected, as compared to control cells treated with 0 µM H_2_O_2_ (**Figure 2A**). For example, the CellTiter-Blue value was reduced by 12.5% (*p*<0.05 vs control) and 12.75% (*p*<0.05 vs. control) of untreated control cells after 18 h exposure to 200 µM and 500 µM H_2_O_2_, respectively. The CellTiter-Blue value observed at the 500 µM H_2_O_2_ concentration was not significantly different from that observed at the 100 µM (*p* = 0.802) or 200 µM (*p* = 0.938) H_2_O_2_ treatments. Protein carbonyls are the most widely studied markers of protein oxidation and are frequently used as markers of oxidative stress [29]. To determine the effect of H_2_O_2_-mediated oxidative stress on protein carbonylation, ARPE-19 cells were treated with various doses of H_2_O_2_ for 18 h. Protein carbonyls were measured by colorimetric assay in total protein that was isolated from H_2_O_2_-treated cells. Compared to untreated cells, a significant increase in protein carbonyls was observed in cells treated with H_2_O_2_ (100 µM–500 µM), suggesting that exposure to H_2_O_2_ -mediated oxidative stress produced damage to cell proteins (**Figure 2B**). In the rest of our experiments, 200 µM concentration of H_2_O_2_ was used to assess the H_2_O_2_-mediated effect on gene expression.

### Effect of miR-30b mimics and antagomirs on cell viability

**Figure 3:**
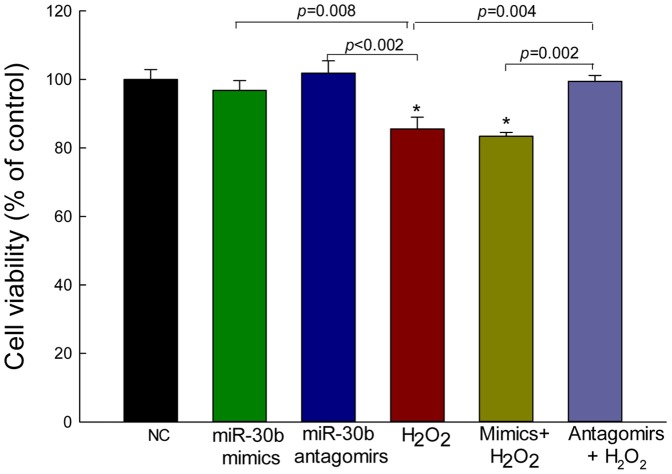
Effect of mimics and antagomirs on cell viability assessed by CellTiter-Blue in ARPE-19. ARPE-19 cells were transfected with mimics (20 nM) and antagomirs (50 nM) in presence or absence of H_2_O_2_ treatment, and incubated for 24 h before adding the CellTiter-Blue reagents; H_2_O_2_ (200 µM) was added for the last 18 h. NC (20 nM) and H_2_O_2_ (200 µM) alone were also used as controls. Data were analyzed using analysis of variance (ANOVA) with Student–Newman–Keuls multiple comparison tests and are expressed as a percentage of the untreated control. The CellTiter-Blue values as labeled by an asterisk are significantly different from the control (*p*<0.05). Values are presented as mean ± SEM; n = 5 per group.

To eliminate the possibility that the inhibitory action of miRNA mimics on catalase expression in presence of H_2_O_2_ was due to the toxic effect of H_2_O_2_, we measured the cellular viability at 24 h and found no significant changes in the cells transfected with either mimics (20 nM) or antagomirs (50 nM) of miR-30b, when compared to negative control (NC). H_2_O_2_ alone and together with miR-30b mimics significantly (*p*<0.05) reduced the cell viability, as compared to NC. H_2_O_2_ alone also showed a significant difference in cell viability when compared with mimics (*p* = 0.008) and antagomirs (*p*<0.002). However, miR-30b antagomirs significantly rescued H_2_O_2_-mediated cell death, as compared to cells treated with H_2_O_2_ alone (*p* = 0.004) or H_2_O_2_ with the mimics (*p* = 0.002) [**Figure 3**].

### Response of miR-30b to H_2_O_2_

**Figure 4:**
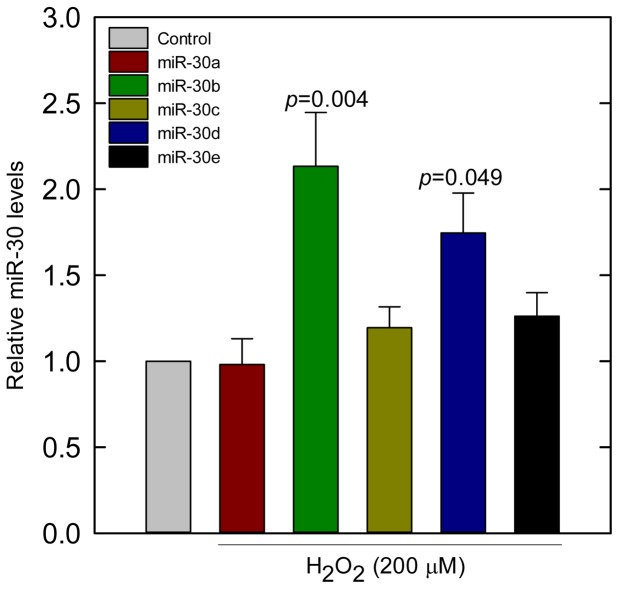
miR-30 targeting human catalase is sensitive to H_2_O_2_. Cultured ARPE-19 cells were treated with vehicle or 200 µM H_2_O_2_ for 18 h before harvesting. The expression levels of miR-30 family members were determined by qRT-PCR using snRNA U5 as an internal control. Values are the means ± SEM of relative changes over controls from four samples in each group after normalization to snRNA U5. H_2_O_2_ increased the expression levels of miR-30b (*p* = 0.004) and miR-30d (*p* = 0.049) miRNAs as compared to the control.

In order to investigate whether the expression of miR-30 family members was influenced by H_2_O_2_, ARPE-19 cells were treated with vehicle or 200 µM H_2_O_2_ for 18 h. The expression level of miR-30b as determined by qRT-PCR was found to be sensitive to H_2_O_2_ (*p* = 0.004) when compared with the control. The expression of miR-30d was also seen to be up-regulated by H_2_O_2_ treatment (*p* = 0.049). The expression of other members of miR-30 family (miR-30a, miR-30c, and miR-30e) was not observed to be altered by H_2_O_2_ treatment, as compared to the control (**Figure 4**).

### Validation of *in silico* target analysis of miR-30b

**Figure 5:**
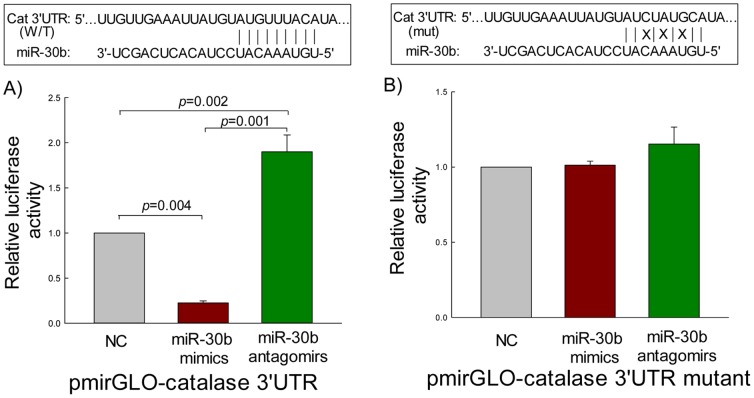
miR-30b interacts with the human catalase 3′UTR. ARPE-19 cells were co-transfected with wild-type (WT, **Figure 5A**) or mutant (mut, **Figure 5B**) catalase 3′-UTR s and 20 nM scrambled miRNA (NC), 20 nM mimics, or 50 nM antagomirs using Lipofectamine™ 2000 transfection reagent. Cells were harvested 24 h post-transfection for measuring luciferase activities by the dual luciferase reporter assay system, as described in ‘Materials and Methods’. The Firefly luciferase activity was normalized with the *Renilla* luciferase activity. The levels of luciferase activity of miR-30b mimics and miR-30b antagomirs groups were compared with those of NC-transfected cells; NC values were set to 1. Values are the means ± SEM of relative luciferase activity over NC after normalization to the *Renilla* luciferase activity from two independent experiments in triplicate. Sequences of wild-type or mutant (3 bp mutation within the seed region) target site for miR-30b in catalase 3′-UTR are shown above the figure. The levels of significance between the groups are shown inside the figure (5A).

To experimentally validate the computational data, a pmiR-GLO luciferase construct with the catalase 3′-UTR was generated. The purified gel product of catalase 3′-UTR was cloned into the cloning site downstream of the luciferase gene as mentioned in ‘Materials and Methods’. A mutant version of pmir-GLO-catalase-3′-UTR-mut with 3 bp mutation within the seed region (**Figure 5B**) was also generated. A significant decrease (*p* = 0.004) in relative luciferase activity was observed when pmir-GLO-catalase-3′-UTR was co-transfected with miR-30b mimics as compared with the scrambled miRNAs (**NC,** **Figure 5A**), and the miR-30b mimics-mediated suppression was abolished by the mutation of the 3′-UTR miR-30b binding site, which disrupts the interaction between miR-30b and the catalase-3′-UTR (**Figure 5B**). miR-30b antagomirs not only restored the wild-type 3′-UTR-modulated luciferase activity, but also significantly increased its activity compared with the NC (*p* = 0.002) and miR-30b mimics (*p* = 0.001) (**Figure 5A**).

**Figure 6:**
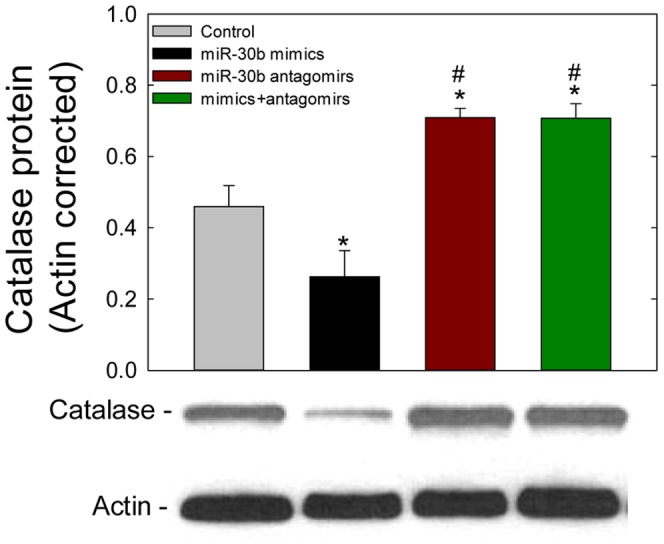
Effects of miR-30b mimics and antagomirs on catalase protein expression in ARPE-19 cells. Cells transfected for 24 h with NC, miR-30b mimics, or miR-30b antagomirs, were lysed and subjected to western blotting following the protocol mentioned in ‘Materials and Methods’. The PVDF membrane was immunoblotted with anti-catalase and anti-μ-actin antibodies. *β*-Actin was used as a loading control. Data reported here are representative of the experiment performed in triplicate. The ratio of band intensity is relative to that of *β*-actin. The band intensity was measured by using ImageJ software (see ‘Materials and Methods’). Values are presented as mean ± SEM; n = 3. *p<0.05 vs. control, # *p*<0.05 vs. miR-30b mimics.

The Western blotting analyses further confirmed the luciferase assay results. Here, transfection with the mimics of miR-30b resulted in decrease of catalase protein expression (*p*<0.05) when compared with the control, whereas the antagomirs of miR-30b protected the miR-30b mimics-mediated inhibition of catalase expression (*p*<0.05) even in presence of miR-30b mimics (*p*<0.05) (**Figure 6**).

### Antagomirs of miR-30b protects miR-30b-mediated suppression of catalase expression under oxidative environment

**Figure 7:**
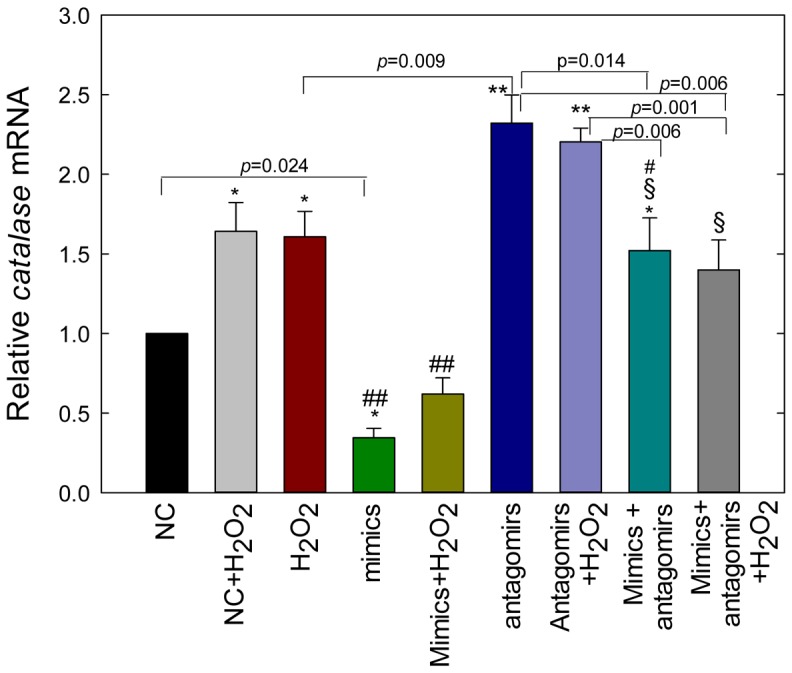
Antagomirs of miR-30b protect ARPE-19 cells from miR-30b mimics-mediated inhibition of catalase expression under oxidative condition. Cells were transfected with NC (20 nM), mimics (20 nM), antagomirs (50 nM), or mimics+antagomirs, and incubated for 24 h before harvesting for total RNA extraction; H_2_O_2_ (200 uM) was added for the last 18 h. Catalase mRNA levels were expressed relative to *Hprt* mRNA with the value of the scrambled miRNA-transfected control (NC) set to 1. Values are presented as mean ± SEM; n = 4. *p<0.05 vs. control (NC), **p<0.001 vs. NC/mimics/mimics+H_2_O_2_/mimics+antagomirs+H_2_O_2_, §*p*<0.001 vs. mimics/mimics+H_2_O_2_, # *p*<0.05 vs. antagomirs, ## *p*<0.001 vs. H_2_O_2_/NC+ H_2_O_2_.

To determine the protective effect of antagomirs on catalase expression under oxidative stress, ARPE-19 cells were transfected with scrambled miRNA (NC, 20 nM), mimics (20 nM), antagomirs (50 nM), or mimics and antagomirs together, and then the cells were exposed to oxidative stress (**Figure 7**). H_2_O_2_ in presence or absence of NC significantly increased the level of catalase mRNA expression (*p*<0.05) as compared with the control (NC). miR-30b mimics not only significantly inhibited catalase expression (*p*<0.05) when compared with the NC, but also very significantly (*p*<0.001) suppressed the H_2_O_2_-stimulated expression of catalase. However, what is interesting is that miR-30b antagomirs not only protected miR-30b mimics-mediated inhibition of catalase expression (*p*<0.001), but also significantly (*p*<0.001) enhanced its expression even when cells were stressed with H_2_O_2_. Furthermore, mir-30b antagomirs-mediated increase of catalase expression was significantly higher than the cells treated with H_2_O_2_ (*p* = 0.009). However, co-transfection of miR-30b antagomirs together with the mimics of miR-30b in presence or absence of H_2_O_2_ resulted in significant increase of catalase expression as compared with the NC (*p*<0.05) or with the groups mimics/mimics+H_2_O_2_ (*p*<0.001).

## Discussion

It is known that H_2_O_2_ induces cell death through an apoptotic cell death mechanism [30], and higher doses of H_2_O_2_ (>1 mM) were demonstrated to activate the Rac1/JNK1/p38 signaling cascade and mediate nuclear translocation of apoptosis-inducing factor (AIF)/Bax into the nucleus, which leads to H_2_O_2_-induced apoptosis [31]. Also, the exposure of H_2_O_2_ (<300 µM) in ARPE-19 cells was reported to increase the cytochrome *c* and AIF contents in the cytoplasm that leads to Fas-regulated Apoptosis [32]. In our experiments, we decided to use 200 µM H_2_O_2_ to induce oxidative stress, since cells treated with H_2_O_2_ in this concentration in our preliminary experiment produced a moderate amount of oxidative stress, as compared to the control. We also measured the levels of protein carbonyls, which serve as a biomarker for cellular oxidative protein damage [33], in ARPE-19 cells treated with various concentrations of H_2_O_2_, and found that the levels of protein carbonyls were significantly increased in cells exposed to 100–500 µM H_2_O_2_, as compared to untreated cells. H_2_O_2_ was also shown to induce a dose-dependent reduction in cellular oxidative capacity. As compared to the control, a significant reduction in oxidative capacity was detected, even when the cells were exposed to 100 µM H_2_O_2_ for 18 h. However, 200–500 µM H_2_O_2_ did not generate much resorufin fluorescence as compared to untreated control cells, because the high catalase activity in RPE cells might have catalyzed H_2_O_2_ very efficiently [5], [34]. Also, increased expression of the stress proteins, Hsp27 and Hsp70, might have enhanced the protective ability of RPE cells against H_2_O_2_-induced stress [35].

In our investigation, we found that the expression of miR-30b and miR-30d was sensitive to H_2_O_2_ stimulation. This finding is not consistent with the work done in rat cardiac cells, where miR-30b and miR-30d expression was reported to be reduced by H_2_O_2_ mediated-oxidative stress [36]. However, hypoxia was found to up-regulate the expression of miR-30b in many cell types [37]. Also, the expression of miR-30d was significantly up-regulated in metastasized hepatocellular carcinoma tissues [38]. Therefore, the response of miRNAs to H_2_O_2_ is probably cell-type specific.

The H_2_O_2_ treatment in our experiment did not influence the expression of the other three members (miR-30a, miR-30c, and miR-30e) of miR-30 family. Although the mature miRNA sequences of all the members of miR-30 family share a common 8-mer conserved seed sequence, the flanking sequences between the members are substantially different from each other (**Figure 1A**). The molecular mechanism of ROS-mediated gene regulation of the miR-30 family members has not been demonstrated yet. It is possible that the mechanisms involving the epigenetic regulation at the DNA level [13] and transcription factor at the transcriptional level [17] regulate the genes that mediate the transcription of miR-30b and miR-30d. The transcriptional regulation of the members of miR-30 family seems to be different from each other, since they are regulated by different promoters of their respective genes. The miR-30 family is comprised of five distinct mature miRNA sequences, which are organized into three clusters: miR-30a/miR-30c-2, miR-30d/miR-30b, and miR-30e/miR-30c-1 [39]. Our *in silico* analyses demonstrated that miR-30c and miR-30e are localized in the intron of nuclear transcription factor Y, gamma (NF-YC) on chromosome 1. miR-30a derived from the intronic sequence of ‘chromosome 6 ORF155’, a putative transcription factor, is located on chromosome 6. miR-30b and miR-30d are possibly clustered and expressed under the control of the promoter of ZFAT (zinc finger and AT hook domain containing) gene located on the chromosome 8. The sensitivity of miR-30b/miR-30d to H_2_O_2_, as demonstrated in our experiment, is possibly mediated through the transcriptional regulation of the promoter of ZFAT gene. In the future, the promoter analysis of the gene of miR-30b/miR-30d is expected to reveal the mechanism of the ROS-mediated gene regulation of miR-30b/miR-30d.

As shown in **Figure 1**, all five members of the miR-30 family (miR-30a through miR-30e) have one 8 bp conserved target site in catalase 3′-UTR. It was reported that binding sites with as few as 7 bp of complimentarity (seed sequence) to the miRNA 5′end are sufficient to confer regulation *in vivo* [40], [41]. Moreover, absence of G∶U wobble pairing in the seed sequence and the substantial 3′ pairing of miR-30 with the catalase 3′-UTR strongly led us to believe that the catalase 3′-UTR could be a target of miR-30. G∶U wobble pairing in the seed sequence is reported to be detrimental to miRNA function [41]. To determine the potential role of miR-30 in H_2_O_2_-mediated cellular effects on antioxidative defense system in ARPE-19 cells, we selected a H_2_O_2_-upregulated miRNA, miR-30b. *In silico* analysis demonstrates that miR-30b can target a number of genes, including the human catalase. In our experiments using ARPE-19 as a human RPE model, we are for the first time demonstrating that miR-30b is able to bind to the human catalase gene and regulate its expression. This *cis*-regulation occurs by direct interaction of miR-30b through the perfect match of its seed sequence to the binding site in the catalase 3′-UTR and that interaction in our experiment was destroyed when the miR-30b binding site was mutated by three nucleotides. In addition to *cis*-regulation (direct targeting of the mRNA and induce its degradation or inhibit protein translation), miRNA may alter the expression of transcription factors or other regulatory genes that can affect the regulation of the target gene through transregulatory mechanism [42]. However, it is not known whether miR-30b could regulate human catalase expression through miRNA-mediated *trans*-regulatory mechanisms.

In our experiment, functional analysis of miR-30b using specific mimics validated its role in targeting the catalase mRNA. The mimics of miR-30b significantly reduced intracellular expression of catalase while an inhibitor of miR-30b (miR-30b antagomir) protected ARPE-19 cells from oxidative damage by increasing the levels of catalase (**Figure 7**). The reduction of catalase expression in ARPE-19 cells transfected with miR-30b mimics would suggest that the action of miR-30b is mediated through degradation of catalase mRNA, which in turn reduced the abundance of catalase protein (**Figure 6**). By employing luciferase vector with the cloned target 3′-UTR region of catalase mRNA, we also demonstrated that the negative effect of miR-30b on catalase levels in the ARPE-19 cell was the result of direct targeting of catalase mRNA by miR-30b. It was also shown by others that the binding and degradation of the target mRNA levels despite partial base pairing is one of the important mechanisms of action by which miRNAs reduce the levels of their targets in the cell [18], [43]. However, the turnover of catalase protein by miR-30b if mediated separately at the translational level needs to be studied.

To minimize the adverse effects of ROS, cells evolved numerous antioxidant defenses, including catalase. In our investigation, it was observed that ARPE-19 cells exposed to H_2_O_2_ increased the expression of miR-30b, which in turn inhibited the expression of cellular catalase.

We have also found in ARPE-19 cells that H_2_O_2_ has increased the expression of catalase mRNA, which serves as a protective mechanism to decrease the cytotoxicity caused by H_2_O_2_ and other ROS. This compensatory mechanism of an increase of cellular catalase that is associated with an oxidative stress in RPE has been previously reported [34]. However, the mechanisms by which ARPE-19 cells sense H_2_O_2_ and activate catalase expression are not known. In prokaryotes, a number of transcriptional factors including OxyR that regulate the expression of antioxidant genes are reported [44]. In higher eukaryotes, oxidative stress responses are more complex and the molecular mechanisms underlying this phenomenon are not clearly understood. However, many studies point to the involvement of nuclear factor μB (NF-μB) and activator protein-1 (AP-1) transcription factors in the regulation of oxidative stress response [45], [46], and a human study demonstrated that the catalase expression is strongly regulated by the antioxidant-responsive transcription factors Sp1 and CCAAT-recognizing factors in response to oxidative stress response [47]. The implication of transcription factors such as AP1, Sp1 or any other antioxidant responsive element in the regulation of catalase in ARPE-19 cells need to be further explored.

The ROS-mediated oxidative damage plays an important role in the pathogenesis of AMD [48], [49], diabetic retinopathy [50], glaucoma [51], cataractogenesis [52], and other neurodegenerative diseases [53], [54]. The cellular defenses are considered to be important in protecting the RPE cells from the ROS-mediated oxidative stress. To combat the deleterious effect of ROS, cells have evolved a multitier endogenous antioxidant defense system, including the catalytic intracellular catalase. The RPE is reported to contain high amounts of superoxide dismutase [5] and catalase [34]. In our experiments, we found that the transfection of miR-30b mimics or antagomirs did not have negative effects on ARPE-19 cell viability. Rather, mir-30b antagomirs significantly protected the cells from H_2_O_2_-mediated cell death. Also, the transfection of miR-30b antagomirs into ARPE-19 cells not only protected the cells from the miR-30b mimics-mediated suppression of catalase both at mRNA and protein levels, but also significantly enhanced its expression even under oxidative environment. Increased production of the components of the antioxidant defense network is a demanding approach for the treatment of retinal diseases in which oxidative stress-mediated injury plays an important role. Among the components of the endogenous antioxidant defense system, catalase plays a very crucial role in maintaining the redox balance in the cell by detoxifying H_2_O_2_ radicals. Therefore, catalase deficiency creating a redox imbalance of the cells may result in many pathological conditions. It is reported that both untreated and phenytoin (ROS-initiating antiepileptic drug)-exposed acatalasemic (aCat) mice exhibited a 30% increase in embryonic DNA oxidation and a >2-fold increase in embryopathies, as compared to wild-type catalase-normal controls. However, the oxidative stress-mediated disease condition was completely recovered by exogenous catalase [55]. Also, the recombinant adeno-associated virus-mediated transfer of the human catalase gene in the optic nerves of SJL/J mice with encephalomyelitis was found to reduce demyelination by 38%, optic nerve head swelling by 29%, cellular infiltration by 34%, disruption of the blood–brain barrier by 64%, and *in vivo* levels of H_2_O_2_ by 61% [56], and the adenovirus-mediated delivery of catalase to RPE cells *in vitro* and *in vivo* also protected RPE and the neighboring photoreceptors from oxidative stress [57]. Furthermore, overexpression of human catalase in the mitochondria in transgenic mice with a C57BL/6J background reduced H_2_O_2_ production and H_2_O_2_-induced oxidative damage, delayed cataract development, and increased its longevity [58].

In summary, all five members of the miR-30 family are expressed in human RPE cells of which miR-30b and miR-30d are found to be sensitive to H_2_O_2_. Our *in vitro* experiments showed that miR-30b binding to the 3′-UTR inhibits the expression of catalase both at mRNA and protein levels. The antisense of miR-30b (miR-30b antagomirs) protected the RPE cells from the miR-30b mimics-mediated suppression of catalase expression under H_2_O_2_-mediateed oxidative stress. Our study suggests that miR-30b antagomirs could be a useful therapeutic tool to treat the oxidative stress-mediated ocular diseases *in vivo*.

## Materials and Methods

### Cell culture and transfection

ARPE-19 cells purchased from ATCC (Manassas, VA) were grown in Dulbecco's modified Eagle's medium and Ham's F12 medium (DMEM/F12) supplemented with 10% FBS (Hyclone, Logan, Utah), 100 U/ml of penicillin, and 100 µg/ml of streptomycin (Invitrogen, Gibco, Carlsbad, CA). Cells were maintained in an incubator at 37°C under a humidified 5% CO_2_: 95% air atmosphere. The media were changed twice a week. Synthesized RNA duplexes of miR-30b mimics and miR-30b antagomirs were purchased from Qiagen (Valencia, CA). ARPE-19 cells were seeded in 12-well plates at 1.5×10^5^ cells/well and cultured for 48 h and then transfected with scrambled miRNA (NC, 20 nM), miR-30b mimics (20 nM), miR-30b antagomirs (50 nM), or mimics and antagomirs together using Lipofectamine 2000 reagent and OPTI-MEM I reduced serum medium (Invitrogen Life, Technologies, Carlsbad, CA), according to the manufacturer's protocol, and further incubated for 24 h before harvesting for RNA and protein analyses. The effects of mimics or antagomirs of miR30b on mRNA expression were also assessed in cells against H_2_O_2_ (200 µM) exposure. In this group, ARPE-19 cells were treated with mimics or antagomirs of miR-30b for 6 h prior to H_2_O_2_ insult for 18 h and then harvested for total RNA extraction. Control cells without H_2_O_2_ and mimics/antagomirs treatments were also maintained.

### Determination of Cell Viability

The effects of H_2_O_2_, mimics, and antagomirs on the viability of ARPE-19 cells were assessed using a CellTiter-Blue assay (Promega Corp, Madison, WI). The assay measures the ability of living cells to reduce a redox dye (resazurin) into a fluorescent dye (resorufin). Nonviable cells that lose metabolic capacity do not generate a fluorescent signal. The assay was performed according to the manufacturer's protocol. In brief, 96-well plates were seeded at 1×10^4^ cells/well and incubated for 6 h for cells to attach to the surface. Cells were then exposed to varying concentrations of H_2_O_2_, followed by incubation for 18 h. Dilutions of H_2_O_2_ were made fresh from a 30% stock (Sigma-Aldrich, St. Louis, MO) solution into DMEM/F12 media to produce a range of final concentrations prior to each experiment. After washing with DMEM-F12, 100 µL of DMEM-F12 without serum was added to each well, followed by the addition of 20 µL CellTiter-Blue reagents. The plates were incubated at 37°C for 2 h. The fluorescence was then recorded at 560/590 nm in the Synergy 2 Multi-Mode Microplate Reader (Winooski, VT). The cell viability (%) was calculated relative to the positive control (cells not treated with H_2_O_2_). The effect of mimics and antagomirs of miR-30b on cell viability was measured by incubating ARPE-19 cells with 20 nM and 50 nM of mimics and antagomirs of miR-30b, respectively, in presence or absence of 200 µM H_2_O_2_ for 24 h before the addition of CellTiter-Blue reagents. In the group with H_2_O_2_, the cells were incubated with mimics or antagomirs for 6 h prior to H_2_O_2_ treatment for 18 h and then incubated further with CellTiter-Blue reagents for 2 h. The scrambled miRNA (NC) and H_2_O_2_ alone were also used as controls.

### Protein carbonylation assay

Protein carbonyl contents from ARPE-19 cells were measured spectrophotometrically according to commercial kit instructions (protein carbonyl assay kit; Cayman Chemical company, Ann Arbor, MI) with slight modification using a 96-well format. Briefly, ARPE-19 cells treated with varying concentrations of H_2_O_2_ for 18 h were scraped and collected by centrifugation at 2000×g for 10 min at 4°C. The cell pellets were sonicated on ice in 1 ml cold buffer [50 mM MES, pH 6.7, 1 mM EDTA, and protease inhibitors (Sigma-Aldrich, St. Louis, MO)], centrifuged at 10,000×g for 15 min at 4°C, and the supernatant was saved. To remove contaminating nucleic acids, 1% streptomycin sulphate was added to the supernatant and incubated at RT (room temperature, ∼22°C) for 15 min. 800 µl of 2.5 mM DNPH (2,4-dinitrophenylhydrazine) was dissolved in HCl and mixed with 200 µl of sample. A control with equal amount of sample (200 µl) and 800 µl of 2.5 M HCl without DNPH was also used. Control and DNPH-treated samples were incubated in the dark for 1 h at RT. The tubes were vortexed briefly every 15 min during the incubation. After the incubation, 1 ml of 20% Trichloroacetic acid (TCA) was added to each tube, vortexed, incubated on ice for 5 min, and centrifuged at 10,000×g for 10 min at 4°C. The pellet was resuspended in 1 ml of 10% TCA, incubated on ice for 5 min, and centrifuged at 10,000×g for 10 min at 4°C. The pellet was washed twice with 1 ml of (1∶1) ethanol/ethyl acetate mixture, vortexed, and centrifuged at 10,000×g for 10 min at 4°C. After the final wash, the protein pellets were resuspended in 500 µl of guanidine hydrochloride. After centrifugation at 10,000×g for 10 min at 4°C, 220 µl of the samples including the control were transferred to a 96-well plate. The absorbance was determined at a wavelength of 385 nm. The protein contents of the samples on the final pellets after the washes were determined at 280 nm and the carbonyl contents [(carbonyl nmol/ml)/(protein mg/ml)] of cell lysates were calculated from a bovine serum albumin (BSA) standard curve following the company's instruction.

### Gene target analyses

*In silico* analysis of the putative miR-30b that target human catalase were performed using TargetScan (http://www.targetscan.org/) and miRanda (http://www.microrna.org/) algorithms.

### Construction of the vector and luciferase reporter assay

In order to get unidirectional ligation into the vector, we cloned 466 bp of human catalase 3′-UTR containing the one 8 bp target site (5′-ATGTTTAC-3′) for miR-30b into the XhoI-SalI sites downstream of the *luc2* gene in pmirGLO Vector (Promega, Madison, WI) using the following primers: sense 5′-AA*TGAG* **CTCGAG**CGAAGCTTAGCGTTCATCCGTGT-3′; antisense 5′-AA*CATC* **GTCGAC**TTAAGCCATGACGGTGCTCAAG-3′. Bold letters in the primers show XhoI and SalI sites. Both the pmirGLO vector and the purified PCR product of the catalase 3′-UTR were digested with XhoI and SalI (Promega, Madison, WI), and ligated by following the manufacturer's protocol (Promega, Madison, WI). The plasmid containing mutant catalase 3′-UTR (pmirGLO-Cat-3′-UTR-mut) was generated using QuikChange Site-Directed Mutagenesis Kit (Stratagene, Santa Clara, CA) by changing the core of the three miR-30 binding sites from 5′-TGTTTAC-3′ to 5′-TCTATGC-3′.

The PCR parameters for *in vitro* mutagenesis were as follows: 94°C for 3 min, 40 cycles of PCR at 94°C for 30 s, 56°C for 30 s and 72°C for 30 s, 72°C for 7 min and 4°C for 10 min. The wild-type (WT) and mutant cloned 3′-UTR in the vector were verified by sequencing (Genewiz, Inc, South Plainfield, NJ). For the luciferase assay, ARPE-19 cells (3×10^5^/per well) in 6-well plates were transiently co-transfected with 150 ng/well of WT or mutant pmirGLO-Cat-3′-UTR reporter vectors and scrambled miRNA (NC, 20 nM), miR-30b mimics (20 nM) or antagomirs (50 nM) using Lipofectamine 2000 reagent (Invitrogen, Carlsbad, CA). The *Renilla* luciferase-neomycin resistance cassette (*hRluc-neo*) was used as the internal control of the luciferase assay. The luciferase activities were analyzed 24 h later by the Dual-Luciferase® Reporter Assay System (Promega, Madison, WI) according to the company's instructions, using a luminometer (Turner Designs TD20/20, Sunnyvale, CA). The luciferase assay results were expressed as relative firefly luciferase activity over the NC after normalization to the *Renilla* luciferase activity.

### Quantitative RT-PCR (qRT-PCR)

The miRNA-enriched total RNA was extracted from cultured ARPE-19 cells using QIAzol lysis reagent and miRNeasy kit following the protocol of the manufacturer (Qiagen Inc., Valencia, CA). For the reverse-transcription reaction, components including miScript RT buffer, miScript reverse transcriptase mix, and template RNA (100 ng or 10 ng for mRNA and miRNA analyses, respectively) were incubated at 37°C for 30 min followed by incubation at 95°C for 5 min to inactivate miScript reverse transcriptase mix. The qRT-PCR was performed following our previous descriptions [59]. qRT-PCR was performed in MyiQ Cycler (Bio-Rad Laboratories Inc., Hercules, CA) with a 25 µL total volume containing cDNA, 1× SYBR Green PCR Master mix (Qiagen Inc., Valencia, CA), and 300 nM gene-specific forward and reverse primers. The primer sequences used in qRT-PCR analyses of catalase and *Hprt* are as follows: catalase (GeneBank accession number NM_001752.2), forward, 5′-CCATTATAAGACTGACCAGGGC-3′ and reverse, 5′-AGTCCAGGAGGGGTACTTTCC-3′; *Hprt* (GeneBank accession number NM_000194.2), forward, 5′-ACAGGACTGAACGTCTTGCTCG-3′ and reverse, 5′-GTGTGCTCAAGGGGGGCTATA-3′. Primers assay system for miR-30b and *U5 snRNA* were purchased from Qiagen (Valencia, CA). The PCR amplification conditions were the following: 5 min at 95°C, 40 cycles at 95°C for 10 s and 60°C for 30 s. Each sample was assayed in duplicate. The qRT-PCR data were normalized to the expression levels of the housekeeping genes *Hprt* and *U5 snRNA* for mRNA and miRNA analyses, respectively. The specificity of the PCR amplification was confirmed by 1% agarose gel electrophoresis. The expression levels of mRNA and miRNAs were measured by the fluorescence threshold value (C_t_) using MyiQ cycler software (Bio-Rad Laboratories, Hercules, CA). The C_t_ is the fractional cycle number at which the fluorescence of each sample passes the fixed threshold. Briefly, the average ΔC_t_ of each group was calculated by the following formula: ΔC_t_ = average catalase or miR-30b C_t_ – average of housekeeping gene (*Hprt or U5 snRNA*) C_t_. ΔΔC_t_ was calculated by ΔΔC_t_ = ΔC_t_ of control group – ΔC_t_ of the treated group. The fold change for catalase or miR-30b expression level was calculated using 2^−ΔΔCt^ [60].

### Western Blotting analysis

Protein samples were isolated from the confluent ARPE-19 cells growing on 6-well plates by washing in ice-cold phosphate-buffered saline (PBS) and then lysed in RIPA buffer [50 mmol/L Tris-HCl (pH 8.0), 150 mmol/L NaCl, 100 µg/mL phenylmethylsulfonyl fluoride, 1% NP-40, 50 mmol/L NaF, 2 mmol/L EDTA], supplemented with protease inhibitor cocktail (Sigma-Aldrich, St. Louis, MO). Protein concentrations were determined by the Lowry assay [61]. The Western blotting was performed following our previous descriptions [62]. Proteins were incubated with primary antibodies (anti-β-actin, Sigma-Aldrich, St. Louis, MO, 1∶3000, 42 kDa) or anti-catalase (Santa Cruz Biotechnology, Santa Cruz, CA, 1∶5,000, 64 kDa) diluted in 5% milk PBS–Tween overnight at 4°C. After washing with PBST (1× PBS, 0.05% Tween-20) the membrane was further incubated with the horseradish peroxidases-conjugated anti-goat IgG (sc-2378, Santa Cruz Biotechnology, Santa Cruz, CA) at a 1∶5,000 dilution for 1 h at RT. The membrane was washed 4 times in 15 min intervals with PBST and the target proteins were detected by the enhanced chemiluminescence detection system (GE Healthcare, UK). The chemiluminescence signal was transferred on Blue Lite Autorad Film (ISC BioExpresss, Kaysville, UT) and the developed film was scanned for densitometric analyses (Kodak Molecular Imaging, Rochester, New York). For data normalization of catalase protein, β-actin protein was detected on the same membrane. For β-actin detection, the membrane was stripped with the Restore Western Blot Stripping Buffer (Thermo Scientific, Rockford, IL) and then incubated with the horseradish peroxidases-conjugated anti-mouse IgG as a secondary antibody (sc-2005, Santa Cruz Biotechnology, Santa Cruz, CA) at a 1∶5,000 dilution for 1 h at RT (∼22°C).

### Statistical analysis

Data are expressed as mean ± standard error of the mean (SEM). Statistical analysis among groups was performed by the analysis of variance (ANOVA) with Student–Newman–Keuls multiple comparison test where applicable using SigmaPlot (Systat software, Inc). Statistical significance was set as *p*<0.05.
